# A good life? A good death? Reconciling care and harm in animal research

**DOI:** 10.1080/14649365.2021.1901977

**Published:** 2021-03-23

**Authors:** Emma Roe, Beth Greenhough

**Affiliations:** a School of Geography and Environmental Sciences, University of Southampton, Southampton, UK; b School of Geography and the Environment, University of Oxford, Oxford, UK

**Keywords:** Care, animal research, killing/death, science and technology studies, animal geographies

## Abstract

Laboratory animal science represents a challenging and controversial form of human-animal relations because its practice involves the deliberate and inadvertent harming and killing of animals. Consequently, animal research has formed the focus of intense ethical concern and regulation within the UK, in order to minimize the suffering and pain experienced by those animals whose living bodies model human diseases amongst other things. This paper draws on longitudinal ethnographic research and in-depth interviews undertaken with junior laboratory animal technicians (ATs) in UK universities between 2013 and 2015, plus insights from interviews with key stakeholders in laboratory animal welfare. In our analysis, we examine four key dimensions of care work in laboratory animal research: (i) the specific skills and sensitivities required; (ii) the role of previous experiences of animal care; (iii) the influence of institutional and affective environments and (iv) experiences of killing. We propose that different notions of care are enacted alongside, not only permitted levels of harm inflicted on research animals following research protocols, but also harms to ATs in the processes of caring and killing animals. Concluding, we argue for greater articulation of the coexistence of care and harms across debates in geography about care and human-animal relations.

## Introduction

There is increasing regulatory and normative interest in care practices and values that sit at the intersection of both human and nonhuman animal wellbeing. For example, the proposed integration of ‘One Welfare’ into the ‘One World, One Health’ agenda aims to support the implementation of animal welfare standards by emphasizing the link between animal welfare and human wellbeing (Garcia Pinilos et al., 2016). This somewhat utopian vision of the possibility of mutual flourishing can be contrasted with the more cautionary approach offered by work in the environmental humanities (Rose et al., 2017; Van Dooren, 2014a) and critical feminist kinship studies (Govindrajan, 2018; Haraway, 2008), which draws attention to the inescapable violence and death that forms a part of multispecies entanglements. As Haraway puts it, ‘there is no way of living that is not also a way of someone, not just something, else dying differentially’ (2008, p. 80). This is a refrain often ringing in our ears during our studies of caring practices in the laboratory animal research industry: animal technologists (ATs) cannot escape what Levina (2018, p. 247) describes as ‘the irreconcilable conflict at the heart of biomedical research’, namely the juxtaposition of searching for a cure and the harms inflicted to research animals during that process. Levina (2018) suggests that the relationship between humans and laboratory rodents is a duality characterized by ‘conditions of cruel optimism’ (after Berlant, 2011) where the distant hope for a cure is set against the cruelty, immediate suffering, lasting harm and death imposed on research animals.

This duality is echoed by the predominance of a utilitarian framework which shapes the governance of UK animal research, balancing animal harms against human and animal benefits (Animals in Science Committee, 2017). In particular, there has been a considerable focus on minimizing harms to animals through the application of Russell and Burch’s (1959) *Principles of Humane Experimental Technique* and the so-called 3Rs of reduction (in the number of animals used), replacement (of animals with other forms of model, or of some species with other species seen to be less sentient/less capable of suffering) and refinement (of experimental techniques so as to reduce the imposition of distress, suffering and lasting harm on experimental animals). Recently regulators have also shown increasing interest in the culture of the animal facility and how this can support care. According to the EU Directive on Laboratory Animal Care and Welfare (Directive 2010/63/EU, 2013), breeders, suppliers and users of research animals have an obligation to ‘foster a climate of care’ within the institutions where they work. Similarly, the UK Animals in Science Regulation Unit (Animals in Science Regulation Unit (ASRU) (2015b), p. 4, emphasis added) suggests that:

A good culture of care is an environment which is informed by societal expectations of respectful and humane attitudes towards animals used in research. Each establishment will have its own way of conveying its culture of care. However, all establishments are subject to similar governance and legal responsibilities under ASPA [Animals (Science Procedure) Act 1986] to deliver humane care.

As the UK ASRU’s report notes, ‘each establishment will have its own way of conveying its culture of care’ (*ibid*), but unlike the 3Rs it is increasingly recognized that a good culture of care considers how to care for the humans as well as the animals within animal research facilities (Robinson et al., 2020). Drawing on this concern, shared by Levina (2018), we explore how in the space of the animal facility, different notions of care are enacted alongside not only permitted levels of harm inflicted on research animals following research protocols, but also for harms experienced by the ATs who both care for and kill them.

The subjects of our paper are not the researchers or scientists, but laboratory ATs who are closely involved in the breeding and preparation of animals for use in research, and in culling surplus, unwanted animals. In the UK, ATs’ care work is at least in part prescribed through regulatory frameworks. ATs receive extensive training in both animal husbandry and technical procedures to facilitate their compliance with regulation (Animals in Science Regulation Unit, 2015a, p. 12). These regulations set out the responsibilities of personal licence holders, many of whom are ATs, in terms of the care and treatment of experimental animals. These responsibilities include, for example, the provision of food, water and pain relief, monitoring after surgery, and contacting the vet if they have concerns or euthanizing animals if their suffering is perceived to exceed that permitted by the terms of the licence. They may be called upon to carry out licenced experimental procedures such as tail-injections or gavaging (administration of drug or similar directly into the stomach through a tube inserted down the throat). ATs also receive animals back into their care following operations and/or their participation in experiments, and are often called upon to cull animals at the end of experimental procedures or when animals are bred which are surplus to requirements. While this may imply the care provided by ATs is driven by a concern for regulatory compliance, scholars have illustrated how care in the laboratory animal facility also often exceeds that dictated through guidelines, licences and regulations (Druglitrø, 2018; Greenhough & Roe, 2018, 2019); this echoes research with those who provide care – especially family members – for whom the distinctions between caring for and caring about are less clear-cut (Dyck et al., 2005).

Like Levina (2018), we wish to argue that the dual obligation to both care for and kill laboratory animals places ATs in precisely the kind of liminal position described by Berlant (2011) as ‘cruel optimism’, and echoed in the work of animal geographers in fields such as conservation (cf. Crowley et al., 2018). Berlant (2011, p. 24) defines cruel optimism as ‘the condition of maintaining an attachment to a significantly problematic object’, a phrase which in many ways captures the AT’s position vis-à-vis the animals they care for. For Levina (2018), analysing representations of animal research within an industry publication, this was expressed as an irreconcilable conflict between a hope for long-term future benefits to human and animal health and the experimental methods used to get there. In contrast, for the ATs we spent time with, the harms imposed by animal research are measured against the hope of providing good care and a good life for laboratory animals in the here and now. We suggest that in their day-to-day work the ethical calculation ATs perform is not a harm-benefit analysis, but a harm-care analysis, thinking through how the harms imposed on laboratory animals might be mitigated by their care work.

### Contextualising care in laboratory animal studies

Our work is in conversation with a growing body of research in the social sciences and humanities concerned with laboratory animal lives. Moving on from early work on the politics and protests associated with anti-vivisection movements, recently social scientists have begun to focus more explicitly on the practices of animal research, providing insight into the history (Druglitrø, 2016b; Druglitrø & Kirk, 2014; Kirk, 2008; Rader, 2004), present (Birke et al., 2007; Holmberg, 2011; Sharp, 2019) and future (Davies, 2013) of human-animal relations within laboratories. Within this work, questions of what constitutes ‘skilled care’ have become an increasing focus of concern (Druglitrø, 2016a, 2018; Greenhough & Roe, 2011, 2018; Holmberg, 2011), drawing attention to how those who care for laboratory animals become attuned to and entangled with the animals they care for, with implications for both their own and their animals’ wellbeing. This paper continues this work by considering how laboratory ATs address their role in relation to the harms inflicted by research processes, engaging with several dimensions of care scholarship from the fields of social and cultural geography, cultural studies, laboratory animal social science and environmental humanities.

Firstly, our work builds on longstanding interests in social and cultural geography on the giving and receiving of care (Atkinson et al., 2011; Lawson, 2007; Power & Hall, 2018; Raghuram, 2012) by considering care between humans and nonhumans. We propose the relation between humans and laboratory animals offers a particularly interesting point of intersection with normative values of who we should care about (Kearns & Reid-Henry, 2009; Lawson, 2007; Popke, 2006; Raghuram et al., 2009; Smith, 2000), and how this shapes the provision (or not) of care (e.g., Dyck et al., 2005). Laboratory animal care work is a practice where the care needs of human and animal are complicated by deliberate harming. Whilst predominantly care is directed to a human patient (G Davies et al., 2020; Druglitrø, 2018; Gorman & Davies, 2019) whom down-the-line may benefit from a new medical treatment, the welfare of the animal is also an ethical obligation, as, to some degree, is the AT’s wellbeing. In laboratory animal research, ethical scrutiny is usually conducted by a remote committee applying harm-benefit analysis. In contrast, we introduce how ATs seek to counter the harms experienced both by animals they work with, and by themselves (as witnesses to, and sometimes also perpetrators of, those harms), in the immediacy of daily caring. We propose that ATs are practicing what we might term harm-care analysis.

Consequently, our approach when entering the field and interpreting our empirical material is situated in more-than-human approaches to care as ethics in practice within human geography (Gorman, 2019; Greenhough, 2011), as opposed to the principled ethical speculation which can form the basis of discussions of the rights and wrongs of animal research. We find inspiration for this approach in emerging work on care in cultural studies and environmental humanities (Berlant, 2011; Puig De La Bellcasa, 2012, 2017; Van Dooren, 2014b), which shows how the daily capability to care about nonhumans is a complex and situated competency. Building on Druglitrø’s (2018) emphasis on practices of skilled care, we draw out the skills required to negotiate the ethical and emotional landscape such care-work generates (cf. Dam et al., 2020; Navne & Svendsen, 2018). Significantly, we show these care skills emerge from an intimate knowledge of the harms inflicted in the preparation for and execution of research. Our analysis is sensitive to how caring practices are learnt, regulated, maintained or tire; and where the distinction between caring for and caring about is increasingly hard to sustain in contemporary animal research. We find Berlant’s (2004) concept of emotional pedagogies helps us to explore how, for many of those involved, the act of caring for animals in the laboratory is a deliberate ethical choice informed by mixed experiences (both good and bad) of living alongside animal others, where caring can sometimes mean killing, or the opposite. We further consider how the specific ‘storied worlds’ (Van Dooren, 2014a) of the laboratory, and the relations they sustain, are (or at least can be) both supported by the atmospheres within research facilities (what regulators term the ‘culture of care’) and challenged by the very different ways in which those worlds are storied beyond the facility walls.

Considering the broader atmospheres within which care happens also encourages us to locate these everyday acts of caring with respect to the broader infrastructures within which laboratory animal research itself sits. Whilst very different to studies which focus on the provision of care services (Power et al., 2019; Power & Hall, 2018), historians of laboratory animal science have observed the ways in which particular forms of care (most notably those associated with the provision of care labour) are woven into the infrastructures of laboratory animal research (Druglitrø, 2016b; see also Druglitrø & Kirk, 2014; Kirk, 2008). Such work has often focused on how the social and economic infrastructures of laboratory animal science serve to instrumentalise the practice of care (Druglitrø, 2016a; Friese, 2013; Giraud & Hollin, 2016). For example, Druglitrø (2018) emphasizes the role of skilled care in producing good science, and how this was central to the standardization of animal models and their conditions of housing, husbandry and handling in the 1950s. Here, care becomes synonymous with the practical labour of caring for, as opposed to the emotional and ethical labour of caring about, animal wellbeing. However, recent regulatory developments in laboratory animal care mirror institutional moves in human healthcare, extending guidance to encompass competencies to care about (Animals in Science Regulation Unit, 2015a, 2015b), albeit not with quite such measurable outcomes as those used by the NHS ‘culture of care’ barometer (Rafferty et al., 2015). Yet, there remains considerable uncertainty over what a ‘culture of care’ is, how it might be recognized and how to ensure it functions well (G. F. Davies et al., 2016). Furthermore, beyond the walls of research facilities, arguably publics are largely ignorant of ATs’ care work.

Lastly, we examine the effects of the requirement that animal care staff kill those animals they care for. While the prospect of animal death is familiar for those who work with livestock, it is increasingly the norm for the process of slaughter to be carried out at a different site to that of animal rearing and husbandry, and by different people (Miele, 2016; Philo, 1995; Wilkie, 2010), at least within a Western context. Indeed, when large-scale slaughter does take place on the farm, as seen in the UK Foot and Mouth outbreak, it ‘was dramatic, traumatic, and unusual because it was conducted en-masse, on the farm’ (Law, 2015, p. 61). Given that care is positioned as the opposite to imposing harm, whilst norms of imposing animal death are less straightforward and highly situated, we pay particular attention to how care and violence are negotiated alongside each other in the process of choreographing (as Law terms it) a ‘good death’ for research animals in the laboratory, and the consequent impacts on AT wellbeing.

Below we briefly outline our methodology, before examining four key dimensions of care-work in laboratory animal research, and how they might contribute to wider debates in animal and care geographies around the complication of care and harm. Throughout we explore how violence (harm) does not preclude the possibility of care and affection (Govindrajan, 2018).

## Methodology

This paper draws on longitudinal ethnographic research and in-depth interviews undertaken with junior laboratory ATs during their first few years in their job at UK universities between 2013 and 2015, as well as insights from interviews with key stakeholders in laboratory animal welfare. At the start of the research period, each of the researchers spent 2 weeks working alongside ATs in UK universities in order to learn more about what the role entailed and gain insights into workplace culture and conventions. These experiences helped us build relationships with seven junior ATs at the start of their careers. We then followed them over the course of 2 years, interviewing them three times at approximately six-month intervals in order to understand how their approach to their work, and specifically their understandings and practices of care, evolved as they gained experience and undertook professional training in animal technology. What we sought to understand is how ATs at the start of their careers learnt to come to terms with the conflicting requirements to both care for and harm, comfort and kill, the animals they worked with. Additionally, we interviewed key stakeholders (n = 12) in animal technology, including facility directors, NGOs and professional organizations and trainers, and undertook some preliminary interview work at commercial organizations to gain insight into the wider institutional structures and environments within which our participants worked. We obtained written informed consent for all interviews and observation work undertaken, with full disclosure to the communities we visited. To ensure protection of the participants’ identities, all names and institutions in this account have been pseudonymised, and we are unable to give more specific details of fieldwork locations. This research has been reviewed and approved by Queen Mary, University of London, reference QMREC2012/76.

Since that time, we have continued to engage with the UK animal research community, regularly attending and presenting our work at industry conferences and events such as the Institute of Animal Technologists (IAT) Annual Meetings, the Annual Meeting of the Laboratory Animal Science Association (LASA) and FELASA (Federation of European Laboratory Animal Scientists). The findings of this paper have therefore been shared with and refined through conversations with both our participants and the wider animal research community. It is worth noting that interviews with ATs took place before the regulatory impetus to address the ‘culture of care’ (Animals in Science Regulation Unit, 2015a, 2015b) entered the professional animal research community, and thus documents how care was discussed and cultured prior to contemporary initiatives to cultivate institutional cultures of care.

### Skilled care

Our earliest encounters with ATs led us to note how, in the course of their work, they draw not only on established guidelines and protocols, but an almost intuitive sensibility, a capacity to sense and respond to the animals’ needs which we termed ‘somatic senseability’ (after Greenhough & Roe, 2011) or to use Haraway’s (2008) term ‘response-ability.’ Through their training and experiences working with animals, ATs supplement this with ‘species-specific sensitivities developed through time spent with chickens, or monkeys or mice, for example’ (Greenhough & Roe, 2011, p. 55). As an AT describes it:

Basic things we do on like IAT [a professional training course for ATs] and the licence training, so they teach you what you’re looking for in the cage, how the animal should interact with other animals and towards yourself … [but you also] get to know the rooms … you’re learning all the time. (Group AT Interview, 2013)

Through their professional training, an AT might learn the accepted signs of aggressive or self-harming behaviours in mice in theory, but through their day-to-day experience, through spending time with a species, ATs also learn how to sense and alleviate the boredom which might lead to these behaviours:

You give them toys and we give them a nest to play with. And I like to put in some cubes as their food ‘cause then they get a chance to pick that up and play with it … And another good thing to do is put tissue where their water bottle is because they will-, they like to, sort of like, pull off pieces of that and turn it into nest. (Interview with Claire, 2014)

Furthermore, these sensitivities are not only species but strain specific. As Govindrajan (2018, p. 19) argues in work on human-monkey relations, we need to pay attention to the ‘distinctiveness of different kinds’ within the same species. Here we build on earlier work by recognizing the relationships that ATs form are not with mice as a species, but with specific strains of mice. This experience is reminiscent of what multispecies’ scholars term ‘storied worlds’ (Van Dooren, 2014a) which recognize the mutually constitutive experiences of both human and non-human persons. It is these experiences of shared worlds which condition the ways in which these particular humans relate to these particular animals. Such relationships are marked by distinctive social and cultural obligations, and good care here involves both accepting a responsibility for these specific mice (whose distinctive behavioural quirks might themselves be seen as a result of the harms inflicted by genetic alteration or other actions taken to induce or seek to cure disease) and becoming attuned to their specific care needs.

Highly characterized genetically modified mouse models often evidence distinctive behavioural needs and quirks which cannot necessarily be known in advance (Davies, 2012), thus the harm-benefit analysis conducted at a distance will always miss these emergent care needs. Instead, these additional requirements for care become apparent and are managed through the skills and sensitivities of ATs. For example, here an AT describes her response to a ‘flipping’ behaviour by a strain of mouse:

I’ve noticed that if [a certain strain are] by themselves, they constantly flip on their cages, flip and flip and flip and flip. So we’ve got like little igloo houses that we put in there and it’ll stop them. (Interview with Debbie and Fiona, 2013)

Care in this sense is a particular kind of embodied enskillment (Druglitrø, 2018; Greenhough & Roe, 2011), a learnt capacity, which is developed over time through relationships with particular strains. Where you or I might see only a room filled with boxes and boxes of almost identical looking mice, Fiona perceives a stereotypic behaviour – ‘flipping’. ‘Flipping’ is a possible indicator of loneliness, pain, boredom and a capacity for play; the introduction of igloo houses is untested scientifically but is based on her experience. Importantly, in contrast to the remote weighing-up of the harms and benefits of research through processes of peer and ethical review, the countering of practices of harm with care is situated, relational and takes place in the event. It is identified in the time taken to learn how to care for different strains of mice, or the small gestures of comfort (strawberry jelly laced with painkillers, warm mats to rest on, extra bedding) ATs adopt to care for animals following surgery or other procedures. This notion of care as an affective, embodied state is one which resonates with emerging work in the environmental humanities (Puig De La Bellcasa, 2012; Rose et al., 2017; Van Dooren, 2014a). What the study of ATs brings attention to, in particular, is that this affective capacity of caring about a particular animal strain is developed over time – it is not a fixed state, but one which is continually emerging and evolving in response to the harms also inflicted to animals. This relates to our next point.

### Pedagogies of emotion

The capacity to develop somatic sense-abilities is not universal. It entails a particular disposition, actively sought out by animal facility managers and recognized by fellow like-minded ATs. One of our interviewees, a senior manager explains,

’When we interview for junior technicians, having an interest before in animals is very important. If they don’t show an interest, if I say to someone, “Have you got any pets?” and they say “No.” I’m thinking, well are you that interested in animals?’ (Interview with Biological Service Unit Manager, 2013)

It is arguably not fear of breaching compliance that motivates ATs in the care work they perform, but something that emerges generically from their relationships with animals. As one AT puts it, ‘we all really care about our animals’ (Interview with Claire, 2014). Notably, nearly all our junior AT interviewees personally kept pets and had a strong conviction that they had always wanted to work with animals. This presence of animals in their lives continues to feed visceral intuitions about how to emotionally manage living with them. Working with Berlant (2004) we term this as a pedagogy of emotion, the idea that we learn how to feel, and that there is an aesthetics to which objects make you feel fearful, happy, worriedor upset.

Following Berlant (2004), we remember how feelings about animals emerge historically in the individual, in the same way that feelings emerge about race, gender and sexuality. The emotional pedagogy of an animal carer can be informed by different events over the course of a life spent living alongside and caring for animals, for example: the daily commitment to feed, water and exercise/clean-out your pet; rescuing a wild animal that is trapped in the jaws of your pet cat; coming down for breakfast to see that one of the gerbils has died and been partially eaten by the other; stroking the soft-fur of your dying and then dead cat. As well as honing ATs’ somatic sensibilities towards different animal species/personalities, emotional pedagogies help ATs learn to manage the complicated and often contradictory emotions that arise through caring for an animal over its life-course (cf. Schuurmana & Franklin, 2018). Whilst conventional harm-benefit analysis balances the harms imposed on research animals against possible future developments in medical and scientific research, harm-care analysis draws on past experiences when responding to, or actually imposing harm, and seeks to counter it with care. This reflects how care is something negotiated and lived-out in practice (compare Mol et al., 2010), echoing geographical work which positions care as situated and contingent (Bowlby, 2011; Conradson, 2003; Raghuram, 2012).

Importantly, these emotional pedagogies may help ATs cope with the challenges of caring for and killing the laboratory animals with whom they work. Lifelong experiences of caring for animals across different situations necessarily establish an ‘affective realism derived from embodied, affective rhythms of survival’ (Berlant, 2004, p. 11). This learned ‘affective realism’ is evident in one AT’s description of the death of their pet:

It was all the wet weather, you know all the wet weather we had. And guinea pigs are really susceptible to getting like chest infections and stuff. And I just think she picked up a bit of like a chest infection and just sort of like it was so quick […] I didn’t really have time to get to the vets or anything like that […]. It was just one day’s thing. (Interview with Claire, 2014)

’Affective realism’ and ‘emotional pedagogy’ therefore shape ATs’ capacity to work with laboratory animals by both nurturing an inclination to care for animals whilst at the same time offering experiences which accustom them to how such relationships are always conditioned by experiences of death, loss and suffering as well as life, play and flourishing. Interestingly, some ATs actively sought work with laboratory animals because it brings greater scope for animal care than previous roles in pet shops, stables or petting zoos (Interview with Jack 2013; Group AT Interview 2013). Care here is forged in an evolving disposition – folding in their historic experiences with a continual process of learning on the job, which enables ATs to conceptualize and negotiate the juxtaposition of harm with care in different forms and contexts of human-animal coexistence.

### Culturing caring institutions and atmospheres

Given this situated and evolving experience of care, it is unsurprising that understandings of the term ‘culture of care’ vary. In our research, more senior technicians and managers spoke of this in terms of professional standards, embedding within staff a workplace ethic which insists on the highest standards, a strong sense of, as one manager put it ‘how we do things round here’. This understanding of care is one bound-up with the labour of caring for and registered through the markers of professionalism: happy staff, clean and tidy workspaces. For the ASRU, ‘A culture of care at an establishment starts with a culture of [regulatory] compliance’ (Animals in Science Regulation Unit, 2015a, p. 26), echoing arguments in the literature which see care-work in animal research as instrumentalised (Druglitrø & Kirk, 2014; Friese, 2013; Giraud & Hollin, 2016). Within the animal facility however, for the ATs we spoke to, the phrase a ‘culture of care’ captured instead a shared disposition to care about the animals. Furthermore, this disposition was not unique to individuals, but was shared across a site or facility: ‘It’s not just one person who cares’ (Interview with Eleanor, 2013).

These inconsistences in the definition of a ‘culture of care’ – as professionalism, as regulatory compliance and as shared disposition – can be read through Berlant’s (2004, p. 4) work as integral to the broader atmosphere within which care takes place, shaped by ‘different styles of managing simultaneous, incoherent narratives of what’s going on and what seems possible and blocked in personal/collective life.’ Even before the widespread promotion of the culture of care, we found the animal facility to be a space shaped by multiple, simultaneous, incoherent narratives. ATs’ narratives combine the hope of delivering good animal care with a belief in the benefits of biomedical research using animals, and the hope that animal experimentation can be reduced, refined and animals ultimately replaced. Yet ATs also harbour fears: fears about telling people where you work, fears about developing animal allergies, fears about infiltration from animal rights protestors, fears that one has done enough killing of animals in your lifetime, fears that you cannot cope with the animal suffering, fears that the animal you have grown to love must now also die and you will grieve.

While narratives of hope and fear may appeal to a collective culture of care, they perhaps resonate more with atmospheres which condition and shape possibilities for both providing care and coming to terms with imposed harms. These narratives reflect how ATs are, in Berlant’s (2011, p. 15) terms ‘continuously busy judging their environments and responding to the atmospheres in which they find themselves’. The affectivity of the atmosphere within which care is cultured is therefore conditional, shaped by events and relations inside and outside the laboratory, for atmospheres are fluid, fleeting and vulnerable things, both personal and collective, palpable and diffuse (Anderson, 2009; Lorimer et al., 2019). Whilst in contrast to cultures, atmospheres can be engineered and conditioned, more often they are ephemeral and shifting, conditioning and resistant to consolidation. Indeed, ATs in the course of their day-to-day lives transition complex and contradictory atmospheres.

Where a culture of care may be nurtured within research facilities, external challenges from beyond facility walls influence ATs understanding of their relationship to the animals they work with. Laboratory animals, and protests against their use, were front-page news in the UK in the 1980s and 1990s. During that period, anti-vivisectionist perceptions were that there was a dearth of care about animals in the laboratory (Plous, 1991). Direct action and highly visible protests targeting those who worked in animal research led to a situation today where animal research activities are often hidden from public view: buried behind unmarked doors in the basements of research institutes. While recent developments have seen an increased opening up of animal research facilities through public engagement work (Ormandy et al., 2019), there remains a sense of public unease. The complexities we unpack here pose ongoing challenges for those seeking to embed ‘a culture of care’ within wider institutional infrastructures (Hawkins, 2018).

Consequently, while within the animal facility ATs can articulate their hopes and fears without being judged as uncaring, outside the animal facility ATs wrestle with the inability to talk openly about their work and the ethics which underpin it, except perhaps to colleagues, close friends and family. One AT describes below an hostile atmosphere that often refuses to acknowledge her capacity to care:

I just tell them that I’m a research technician for cancer research and I leave it at that really. […] you don’t know how people are going to react to you because there’s still a lot of negative feelings towards animal research. […] I’m not that open with people about what I do ‘cause it’s just-, you can’t trust anyone can you? (Interview with Claire, 2013)

These atmospheres serve to shift attention away from practices of care, emphasizing only the harms inflicted by animal research.

By bringing attention to the atmospheres that shape our understanding of how care is known, practiced and felt, we can recognize situations which constrain and conceal dispositions and practices of caring, and equally those that enhance them. Here we concur with previous work in environmental humanities that has stressed the need to think of care as a situated practice, stressing that ‘we need to ask how to care in each situation’ (Puig De La Bellcasa, 2011, p. 100), but we also ask how is (or is not) care being witnessed and named in narratives when appearing alongside harming?

### Sharing suffering and killing well

In this final section we explore how animal care work is complicated by practices of killing. Within animal welfare science and most policy-making, it is largely accepted that humane ‘death is not a welfare issue’ (Webster, 1994, p. 15), and yet there are voices (Mellor, 2016; Yeates, 2010) who argue that when it excludes an animal experiencing positive living states, death becomes a welfare issue. It is in this complexity that the killing of research animals is normalized as ‘good’ for the animal and science, and yet the intimate life-knowledge ATs have for their animals means this animal welfare debate can undermine killing as straightforwardly acceptable. Consequently, in the midst of killing, different forms of caring can overlap and diverge in complicated ways; as Van Dooren (2014b) suggests in the context of multi-species relations, ‘care for some individuals and species translates into suffering and death for others’. For the ATs we spoke with, these contradictions and complexities play out in the relation with a single animal, one they have cared for (practically given food, water etc.) and cared about (reflected on overall well-being) and are now obliged to kill (as an act of care, whilst perhaps suffering themselves by doing killing).

Birke et al. (2007, p. 100) describe learning to come to terms with animal death as a key rite-of-passage for laboratory ATs. Similarly, Holmberg (2011, p. 157) notes in her ethnographic work on animal research that ‘caring and killing seem to be more closely linked’. This means ATs need to come to terms with the knowledge ‘that your way of being is dependent on the suffering of others (and yourself)’ and then perhaps try to ‘live with that by seeking less painful practices and ways of being’ (Greenhough & Roe, 2010, p. 45; Haraway, 2008). This drive to seek less painful, less fear-ridden practices and ways of being – for both themselves (in terms of the costs and emotional labour of being a laboratory AT (see Davies & Lewis, 2010), and their animals – is very evident in ATs’ discussions of the best methods for culling used or unwanted laboratory animals.

In the extracts below, junior ATs compare the benefits of carbon dioxide (CO_2_) gas and cervical dislocation (breaking the neck) as methods of culling and provide an insight into what, for them, is involved in the process of killing well. Particularly notable in the extract below is the stress they endure witnessing animal death, and efforts to care for themselves through the process (to face the wall).

If you CO_2_ them […] you have to watch them, whereas if you just do their neck, they don’t know it’s coming. But [with CO_2_] you put them in that box and then you just watch them, that’s the horrible thing […]. I normally sit and face the wall and then once it’s gone […] you still have to break their necks after, to make sure that there’s destruction of the brain and stuff, because sometimes they can hold their breath. (Interview with Debbie and Fiona, 2013)

For these ATs, cervical dislocation achieves the best death for both the mice (in terms of minimizing anxiety, pain, distress and excitement) and for themselves (in terms of its aesthetic acceptability). These complexities are reflected in guidance for those working in laboratory animal welfare, which suggests that euthanasia or a ‘good death’ must be,

painless, achieve rapid unconsciousness and death, require minimum restraint, avoid excitement, is appropriate for the age, species, and health of the animal, must minimize fear and psychological stress in the animal, be reliable, reproducible, irreversible, simple to administer (in small doses if possible) and safe for the operator, and, so far as possible, be aesthetically acceptable for the operator. (Close et al., 1997, p. 295)

In practice reconciling these different aspects of a good death is difficult to achieve. The physical and emotional duress of culling, and its articulation here, highlights the complex and often counter-intuitive nature of practicing care for research animals. In seeking a ‘good death’, care is defined and performed in multiple ways across multiple registers (cf. Law, 2015). Firstly, there is the responsibility to protocol, ensuring, as the AT notes ‘the destruction of the brain and stuff’. There is a somatic sensibility which leads one AT to speculate ‘if I had to die would I rather suffocate or would I rather die quickly?’ There is the experience of having lived through other deaths, and the kind of aesthetic realism (after Berlant, 2004) which accompanies and helps ATs to cope (for a while) with the burden of so many deaths:

’culling is part of the job and I knew that when I took it on. But then from working and helping my dad with his sheep […] helping him sort them out going into slaughter […] you learn to deal with those emotions’ (Interview with Claire, 2013).

There is the broader atmosphere of care around the laboratory, nuanced by experiences within and without, including the professionalization of UK-based AT work, the conviction these deaths serve a greater good, the animal rights protestors outside the building and the caution around speaking about one’s work. Finally, there is a commitment to sharing suffering, which for some means ensuring they do the killing because their animals know them, and they would not want a stranger to perform what in some senses might be seen as a final act of care.

Strikingly, at times these complex and contradictory practices of care cannot be resolved. Some days ATs just can’t face the culling, and perhaps ask someone else to help: ‘When I first started as an AT, Seth, [her manager], said: “If you were having a bad day”, [….] if you said, “I can’t do it today,” “That’s fine. You don’t have to cull anything today if you’re not feeling up to it”’ (Interview with Eleanor, 2013). These momentary hesitations counter a simple narrative of laboratory euthanasia as instrumentalised ‘humane’ killing, showing how the relations and infrastructures within the animal facility which appear to be orientated to facilitating animal death can also serve to (momentarily at least) tip the balance and instead make killing impossible.

## Conclusions

This paper began by noting the growing interest in care at the intersection of human and animal wellbeing, the hope for mutual flourishing, and the suggestion that the reality in an animal facility could be more complex, a form of ‘cruel optimism’ (Berlant, 2011). Within the field of animal research, the experiences of ATs delivering research animal care are rarely heard; this paper has addressed that and shown how they do complex and contradictory care-work, countering the harms imposed in the course of scientific research with day-to-day practices of caring for and about the animals they work with. This in turn affects their own wellbeing and emotional competency to care. We have illustrated how ATs learn to accept and deliver care-work where not everything can be cared for (Puig De La Bellcasa, 2012, p. 204) all the time, and thus care-work is necessarily selective and discriminatory; it matters ‘who we are bound up with and in what ways’ (Van Dooren, 2014a, p. 60). Furthermore, our research with ATs stresses how their embodied and emotional care competencies are not pre-given, but developed through a lifetime lived alongside animals. We also showed how these care competency expressions’ can be both nurtured and constrained by local cultures and atmospheres.

We suggest our account of the care-work performed by ATs has much in common with Puig De La Bellcasa’s (2012, p. 197) vision of care as ‘a vital affective state, an ethical obligation and a practical labor’. ATs’ care is the practical work of laboratory animal husbandry, but also obligation that ‘requires that we get involved in some concrete way, that we do something’ (Van Dooren, 2014b, p. no page). ATs, we argue, evidence an emotionally, affective state that counters harms with care for animals, themselves and others, especially witnessed in the empirical discussion of killing and performing a good death. In closing, we wish to make three observations which link our case to wider debates about care, killing and human-animal relations in geography (for an overview see Gibbs, 2020).

Firstly, whilst within animal geography care and killing are often treated separately, our study shows how when care, harm and killing are embedded in the same place, they become situated, co-constitutive practices (Govindrajan, 2018, p. 81). Knowing you (or your community) have inflicted harm on an animal, and will be responsible for its death, affects how you care for it in life. This signals the importance of thinking through not only about how care shapes possibilities for those we care for to flourish, but also how we manage the future prospect that mutual flourishing will come to an end.

Secondly, work on affect and attunement in human-animal relations can teach us much about the process of learning to care about animal others and how to recognize their capacity for agency and experience (Despret, 2004; Druglitrø, 2018; Greenhough & Roe, 2011). It can also serve to explain why we might respond to nonhuman death with feelings of grief and loss (Greenhough & Roe, 2019; Rose et al., 2017), in ways echoed by those who work on experiences of human death (Maddrell, 2016). Here Berlant’s (2011) attention to the ways in which people learn to come to terms not only with the possibilities of mutual flourishing, but also its failure, makes an important contribution shaped not only by the opposition between harm and benefit (Levina, 2018), but the need to counter causing harm with giving care. In this way, the expectation of harm in and of itself offers an incentive to provide the best care possible, at both individual (seen in the care work performed by ATs) and institutional (seen in the increased focus on ‘cultures of care’) scales.

Thirdly, we want to draw attention to how care and (structural) violence are often closely related, in ways which perhaps (or perhaps not) distinguish the case of research animals from much work concerned with care amongst and between human subjects. This has implications for day-to-day life in the laboratory, revealed as a space shaped by the constant physical and emotional labour of seeking to mitigate harm. This careful negotiation of countering harm with care remains challenging to communicate both within and beyond research facility walls, and within an academic tradition in which care and harm are most often seen as diametrically opposed. Whilst the term ‘culture of care’ can embrace these situated, co-constitutive practices, these nuances remain challenging to communicate to wider publics and academic subdisciplines. Recognizing laboratory animal research facilities as a site where care and harm coexist and condition each other is key to building the trust needed between those working in animal research and the wider publics, in order to facilitate a wider appreciation of, and debate about, laboratory animal research. Future work on human-animal (and human-human) relations may benefit from closer attention to the coexistence of care and violence; this also carries broader implications for popular engagements with questions of welfare arguably poorly attuned to how many sites of care also contain (and are conditioned by) the presence of harm.
